# The minor gentamicin complex component, X2, is a potent premature stop codon readthrough molecule with therapeutic potential

**DOI:** 10.1371/journal.pone.0206158

**Published:** 2018-10-25

**Authors:** Westley J. Friesen, Briana Johnson, Jairo Sierra, Jin Zhuo, Priya Vazirani, Xiaojiao Xue, Yuki Tomizawa, Ramil Baiazitov, Christie Morrill, Hongyu Ren, Suresh Babu, Young-Choon Moon, Art Branstrom, Anna Mollin, Jean Hedrick, Josephine Sheedy, Gary Elfring, Marla Weetall, Joseph M. Colacino, Ellen M. Welch, Stuart W. Peltz

**Affiliations:** PTC Therapeutics, South Plainfield, NJ, United States of America; Medical Faculty Mannheim, University of Heidelberg, GERMANY

## Abstract

Nonsense mutations, resulting in a premature stop codon in the open reading frame of mRNAs are responsible for thousands of inherited diseases. Readthrough of premature stop codons by small molecule drugs has emerged as a promising therapeutic approach to treat disorders resulting from premature termination of translation. The aminoglycoside antibiotics are a class of molecule known to promote readthrough at premature termination codons. Gentamicin consists of a mixture of major and minor aminoglycoside components. Here, we investigated the readthrough activities of the individual components and show that each of the four major gentamicin complex components representing 92–99% of the complex each had similar potency and activity to that of the complex itself. In contrast, a minor component (gentamicin X2) was found to be the most potent and active readthrough component in the gentamicin complex. The known oto- and nephrotoxicity associated with aminoglycosides preclude long-term use as readthrough agents. Thus, we evaluated the components of the gentamicin complex as well as the so-called “designer” aminoglycoside, NB124, for in vitro and in vivo safety. In cells, we observed that gentamicin X2 had a safety/readthrough ratio (cytotoxicity/readthrough potency) superior to that of gentamicin, G418 or NB124. In rodents, we observed that gentamicin X2 showed a safety profile that was superior to G418 overall including reduced nephrotoxicity. These results support further investigation of gentamicin X2 as a therapeutic readthrough agent.

## Introduction

The presence of a nonsense mutation in the open reading frame of a gene leads to the introduction of a premature termination codon in the mRNA that results in the production of a truncated-nonfunctional protein product. Nonsense mutations are the cause of approximately 11% of all genetic diseases [1]. Readthrough of premature termination codons to allow production of full length protein has the potential to treat all patients that harbor nonsense mutations as the cause of their disease. We have identified two novel classes of readthrough compounds. The first, ataluren, selectively induces ribosomal readthrough at premature stop codons across many different disease model systems [2–7]. Ataluren demonstrated activity in clinical trials of nonsense mutation Duchenne muscular dystrophy (DMD) [8–10] and has received conditional marketing approval in the European Union for nonsense mutation DMD [11]. The second compound, clitocine, is a nucleoside analog that induces readthrough by incorporating into RNA, including the site of the premature stop codon and has potential therapeutic utility to treat cancers with nonsense-mutated tumor suppressor genes [12].

It is known that aminoglycoside antibiotics can induce readthrough of premature termination codons via modulation of the ribosome in disease causing genes [13–15]. The most common aminoglycosides analyzed in pre-clinical studies of readthrough are gentamicin and G418. Gentamicin is an antibiotic that is used clinically to treat severe Gram negative antibacterial infections despite its potential to induce nephrotoxicity [16] and ototoxicity [17]. In addition to being investigated in many pre-clinical readthrough models, gentamicin has also been investigated in clinical trials of DMD and Cystic Fibrosis (CF) which showed early promising results [18–22]. The aminoglycoside G418 is known to be the most potent and active readthrough compound, and is used extensively in cell-based readthrough assays [23–25]. However, G418 is not used clinically as an antibiotic or as a readthrough agent.

Commercially available gentamicin is composed of a complex of individual aminoglycoside congeners [26–28]. The major components of the gentamicin complex are gentamicins C1, C1a, C2, and C2a constituting 92–99% of the complex. Of the minor components, gentamicin B was found to constitute between 0.8 and 5.3% of the complex, and C2b between 1.3 and 2.1%. Sisomisin was found to be rare, comprising only 0.4–0.6% of the complex. Additionally, gentamicin A and X2 (also known as gentamicin X) together comprised between 1.1 and 7.8% of the mixture. Other components in the complex include gentamicin B1 and garamine.

Here, we investigate the potential to advance an aminoglycoside as a readthrough therapeutic. We report readthrough activity, cytotoxicity and safety of the available gentamicin complex components. We found that all the major components had readthrough activity. One minor component, gentamicin X2, was particularly potent and active. We show in cytotoxicity assays and in tolerability studies in rats that among the aminoglycosides tested, gentamicin X2 may offer the best possibility of achieving a therapeutic window large enough for clinical benefit with limited toxicity.

## Results

### Gentamicin X2, a minor component of the gentamicin complex, is a potent readthrough compound

**Fig 1:**
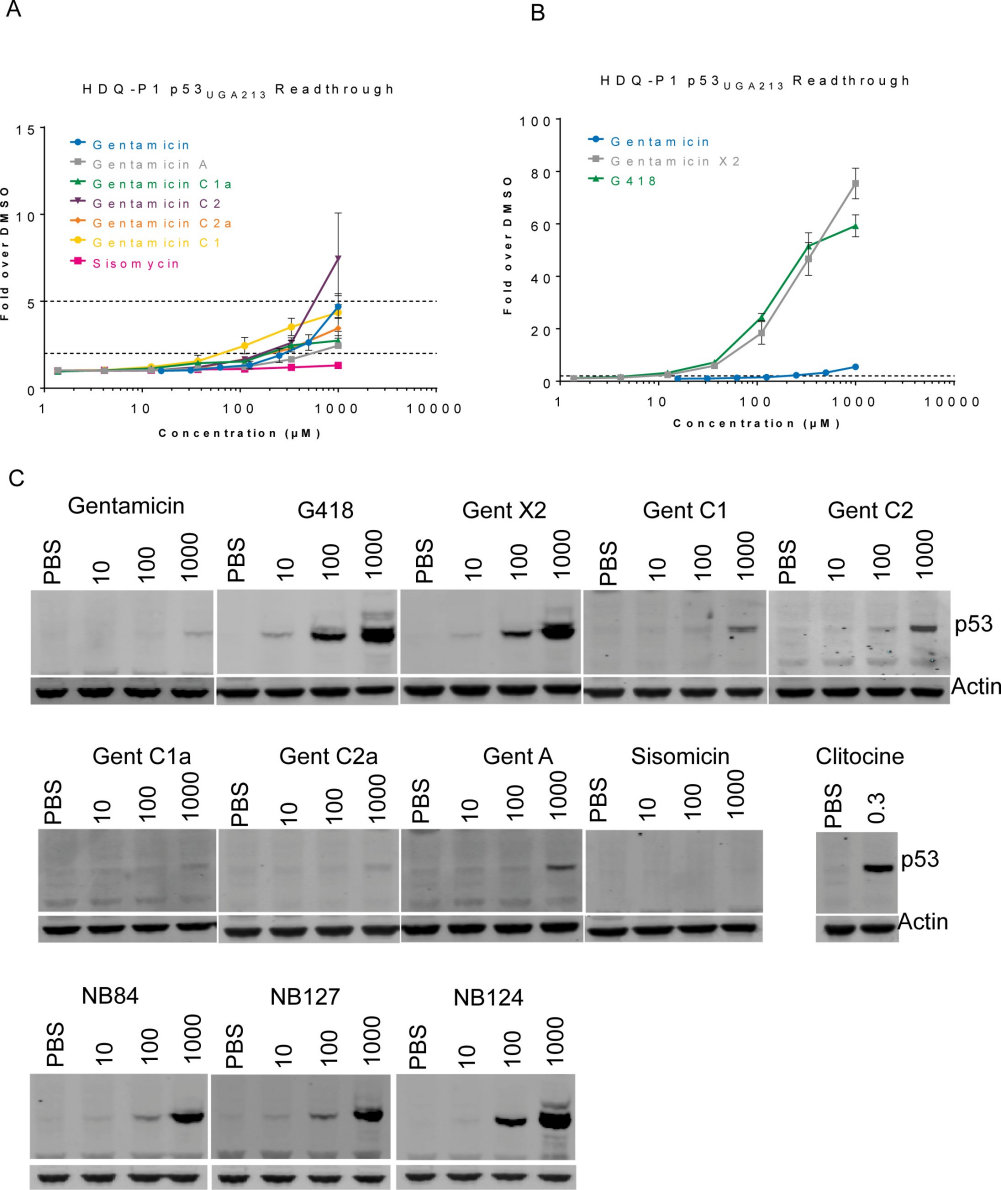
Gentamicin complex and individual components cause readthrough of a native p53-NS allele in HDQ-P1 cells. p53 was detected using a previously described MSD immunoassay (A and B) or by western blot hybridization analysis (C). (A) Readthrough activity of gentamicin complex components is similar to that of the gentamicin complex. (B) Gentamicin X2 and G418 are potent readthrough compounds as compared to the gentamicin complex. Data in Fig 1A and 1B are from a representative experiment. Summarized data from all readthrough experiments can be found in Table 1.

Because it is approved for use as an antibiotic, gentamicin has been investigated as a potential readthrough therapeutic in many different nonsense mutation disease models [29]. In a search to identify therapeutic aminoglycosides with reduced toxicity and increased readthrough potency and activity, we evaluated all of the commercially available gentamicin components and compared their biological activity to that of G418 and the so-called “designer” aminoglycosides NB84 and NB124. We utilized HDQ-P1 mammary carcinoma cells, which are homozygous for R213*, a UGA premature termination codon in the gene for the tumor suppressor protein p53 [30]. Aminoglycoside readthrough activity in HDQ-P1 cells resulted in production of full-length p53 protein that was measured using a previously described p53 Meso Scale Discovery (MSD) immunoassay (**Fig 1A and 1B**) or by western blot analysis (**Fig 1C**) [12;31]. All of the major gentamicin components (C1, C1a, C2, and C2a) were weakly active with EC_2X_ values (the concentration required to double the level of full-length p53 protein) in the triple digit μM range and maximum readthrough activity ranging from 3- to 7-fold above background (**Fig 1A and 1C and Table 1**). In contrast, we observed that among the three minor components available to us (gentamicin A, X2 and sisomycin) gentamicin X2 was the most active readthrough component inducing full length p53 with an EC_2X_ of 19 ± 6 μM and a maximum fold increase above background of 39 ± 31 (**Fig 1B and 1C and Table 1**). The readthrough potency of gentamicin X2 was slightly less than that of the most potent and active aminoglycoside known, G418 (EC_2X_ of 9 ± 5, maximum fold induction of 44 ± 19).

**Table 1:** Aminoglycoside potency and activity in readthrough assays.

Compound	HDQ-p53_UGA213_ (EC_2X_, μM)	HDQ-p53_UGA213_ (Maxfold)	tGFP-542X (EC_2X_, μM)	tGFP-542X (Maxfold)	fLuc-190-NS (EC_2X_, nM)	fLuc-190-NS (Maxfold)
Geneticin (G418)	9 ± 5	44 ± 19	9 ± 4	318 ± 93	6.4 ± 0.2	53 ± 2
Gentamicin X2	19 ± 6	39 ± 31	36 ± 16	135 ± 87	29 ± 9	195 ± 18
NB84	17 ± 5	30 ± 12	38 ± 12	110 ± 57	45 ± 11	162 ± 11
NB124	21 ± 6	62 ± 26	51 ± 25	180 ± 22	25 ± 7	180 ± 23
Gentamicin	418 ± 310	4 ± 1	485 ± 67	4 ± 0.8	1028 ± 89	71 ± 8
Gentamicin C1	311 ± 321	5 ± 3	439 ± 192	3 ± 2	1061 ± 262	19 ± 2
Gentamicin C2	142 ± 86	6 ± 5	266 ± 44	9 ± 2	1086 ± 763	97 ± 12
Gentamicin C1a	228 ± 159	3 ± 1	715 ± 195	2 ± 0.1	1123± 59	19 ± 2
Gentamicin C2a	252 ± 49	4 ± 1	583 ± 110	3 ± 0.5	1077 ± 29	32 ± 5
Gentamicin A	586± 100	3 ± 0.4	385 ± 123	4 ± 0.4	450 ± 15	6.7 ± 0.4
Sisomycin	>1000	1.3 ± 0.1	>1000	1 ± 0.1	2596 ± 428	4 ± 0.5

Values are reported as averages plus or minus the standard deviation for at least three separate experiments. [7;12;31]. EC_2X_: concentration required to double the level of full-length p53 protein. Maxfold: maximum readthrough activity achieved above background. All values used for calculating averages can be found in S1 File. Note: maximum fold values for gentamicin X2 and G418 differ in Table 1 from that in Fig 1B because the highest concentration used for the measurements in Table 1 was 300 μM whereas Fig 1B was 1000 μM.

Because readthrough potency for a given compound can vary between readthrough assays, we wished to compare the rank order of potency of the aminoglycosides in three different readthrough assays–HDQ-p53_UGA13_ and tGFP-542X cell-based assays and fLuc-190-NS cell-free assay (**Table 1**). ThefLuc-190-NS assay is an in vitro rabbit reticulocyte lysate translation of an fLuc mRNA harboring a UGA nonsense mutation at codon 190 (fLuc-190-NS) [7]. It is believed that positively charged aminoglycosides are poorly taken up by cells, and we used this cell-free readthrough assay to eliminate the potential variable of cell uptake when measuring readthrough potency. Note that all the aminoglycosides tested were more potent in the cell-free readthrough assay (**Table 1**). As further confirmation of readthrough potency, the previously described tGFP-542X readthrough assay was used [31]. This assay consists of 293H cells stably expressing a construct containing the coding sequence of tGFP followed by codons 539–545 of human CFTR (with codon 542 being UGA) followed by hemagglutinin (HA-tag) and six histidine (HIS-tag) coding sequences. Readthrough protein was detected using a HA-HIS MSD immunoassay described previously [31].

**Fig 2:**
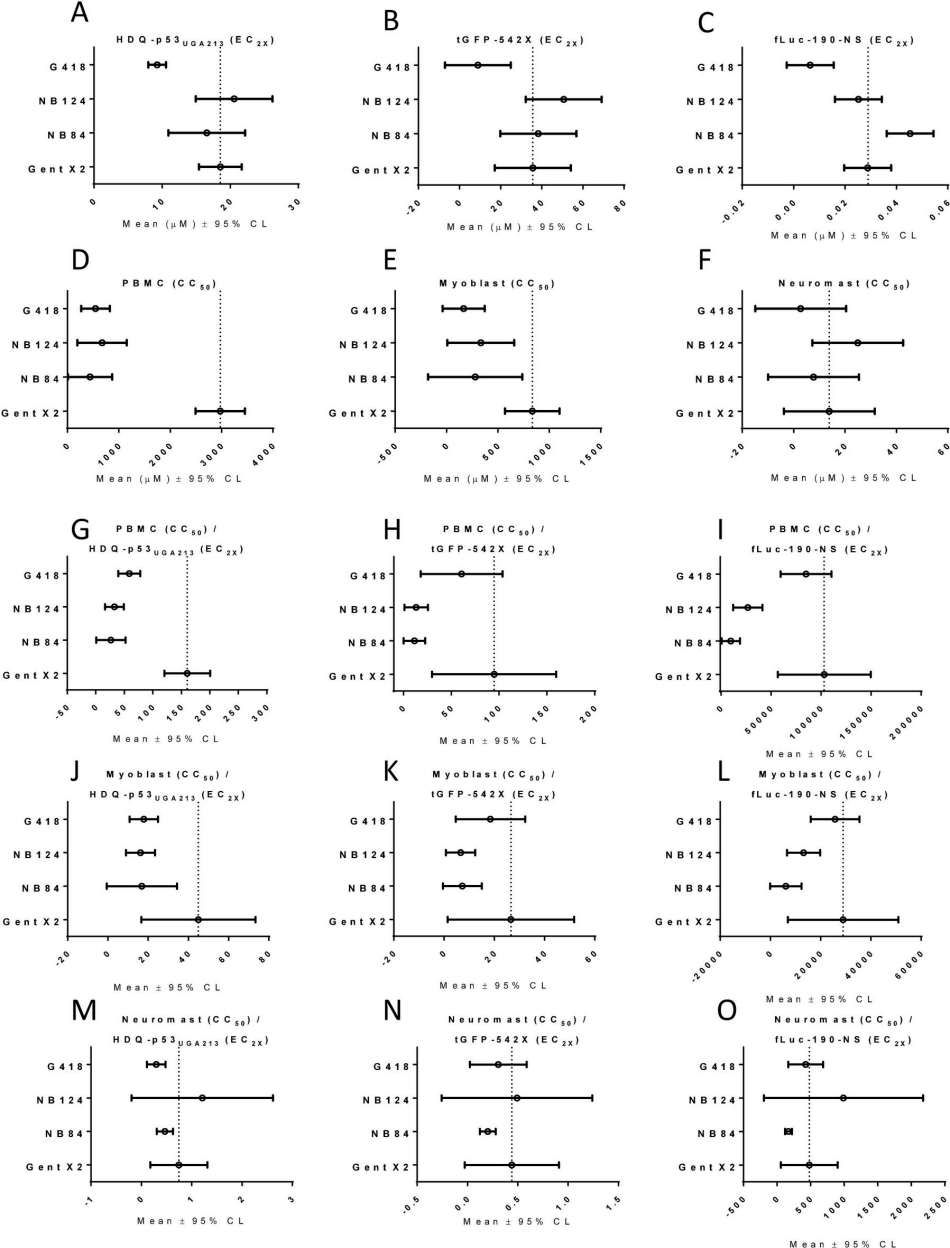
Statistical comparison of selected aminoglycosides. Graphs show average values for at least three independent experiments (open circles) for each aminoglycoside indicated on the y-axis. Error bars indicate the 95% confidence limits for each average. The title of each graph indicates the average measurement or the average calculated ratio. **A-C** are potency averages from the three readthrough assays; **D-F** are averages for the three cytotoxicity assays and **G-O** show the mean ratios between the readthrough and the cytotoxicity assays. All values used for calculating averages can be found in S1 File.

To rank order the compounds, we statistically compared average readthrough potency (EC_2X_) using the 95% confidence limits of the means (**Fig 2A–2C**) [32;33]. Means were considered statistically different if they fall outside of the 95% confidence limit of the comparator mean and vice versa. NB84 and NB124 were equivalent in potency to gentamicin X2 in all the assays except in fLuc-190-NS where NB84 was less potent. In all three assays, G418 was the most potent. The potency rank order was G418 > gentamicin X2 = NB124 = NB84 > gentamicin, with the exception of NB84 being slightly less potent than NB124 and gentamicin X2 in the fLuc-190-NS cell-free assay.

### Gentamicin X2 has a greater readthrough/safety window than gentamicin, G418 or NB124

When considering compounds for potential readthrough therapy it is important to monitor cellular and animal safety. In order to determine the most promising compounds to further evaluate in in vivo safety, we measured cytotoxicity in two different cell types. We used activated human peripheral blood mononuclear cells (PBMCs) (**Table 2 and Fig 2D and 2E**). PBMCs are a primary cell that when activated with phytohaemagglutinin (PHA) expand quickly and are commonly used as an in vitro surrogate for rapidly growing cells in the human body to evaluate cytotoxicity [34]. Prior to advancing compounds into human studies, safety evaluation in rodents is performed. As an in vitro surrogate for rodent toxicity, we used primary mouse myoblasts. We statistically compared average CC_50_s (cytotoxic concentration 50%) using the 95% confidence limits of the means (**Fig 2D and 2E**) [32;33]. Means were considered statistically different if they fall outside of the 95% confidence limit of the comparator mean and vice versa. Statistical comparison of the average CC_50_s) showed that gentamicin X2 was less cytotoxic than G418, NB84 or NB124 in both activated PBMCs and myoblasts (**Fig 2D and 2E**) indicating that gentamicin X2 is a potentially more promising readthrough compound than the other aminoglycosides.

**Table 2:** Aminoglycoside toxicity.

Compound	Stimulated PBMC (CC_50_, μM)	Myoblast (CC_50_, μM)	Neuromast (CC_50_, μM)
Geneticin (G418)	546 ± 286	166 ± 100	2.8 ± 1.2
Gentamicin X2	2974 ± 533	834 ± 623	14 ± 7
NB84	674 ± 265	334 ± 109	8 ± 0.1
NB124	674 ± 265	334 ± 109	15 ± 4

Values are reported as averages plus or minus the standard deviation for at least three separate determinations except for neuromasts where a maximum of two measurements were obtained. All values used for calculating averages can be found in S1 File.

Aminoglycoside antibiotics exhibit well known toxicities to the kidney and hair cells of the inner ear [16;17]. Toxicity of compounds to zebra fish hair cells (neuromasts) is used as a surrogate for potential human inner ear hair cell cytotoxicity [35–38] We evaluated the effect of the compounds on zebrafish neuromasts, and found no statistical differences between the compounds (**Fig 2F, Table 2 and S1 Fig**).

Cellular potency of a small molecule drug will determine the concentration needed for therapeutic effect in vivo. When comparing compounds, the ratio of cellular cytotoxicity to readthrough potency (termed safety window) is a surrogate for in vivo therapeutic window (the ratio between efficacious dose and toxic dose). We calculated safety windows by dividing average cellular cytotoxicity values (average CC_50_s) for all three cytotoxicity assays by readthrough potency (average EC_2X_s) for all three readthrough assays and determined the 95% confidence limits for each ratio in order to statistically compare compounds. Ratios were considered statistically different if they fall outside of the 95% confidence limit of the comparator ratio and vice versa. Gentamicin X2 had a statistically better safety window (based on 95% confidence limits) than either NB84, NB124 or G418 in PBMCs. The safety window in myoblasts for all three readthrough potency measures trended in favor of gentamicin X2, but did not reach significance (**Fig 2J–2L**). Safety windows in neuromasts did not statistically distinguish between the compounds (**Fig 2M–2O)**. Based on the analysis presented in Fig 2 and discussed above we concluded that gentamicin X2 warranted further safety analysis.

### Gentamicin X2 was better tolerated in vivo than was G418

**Fig 3:**
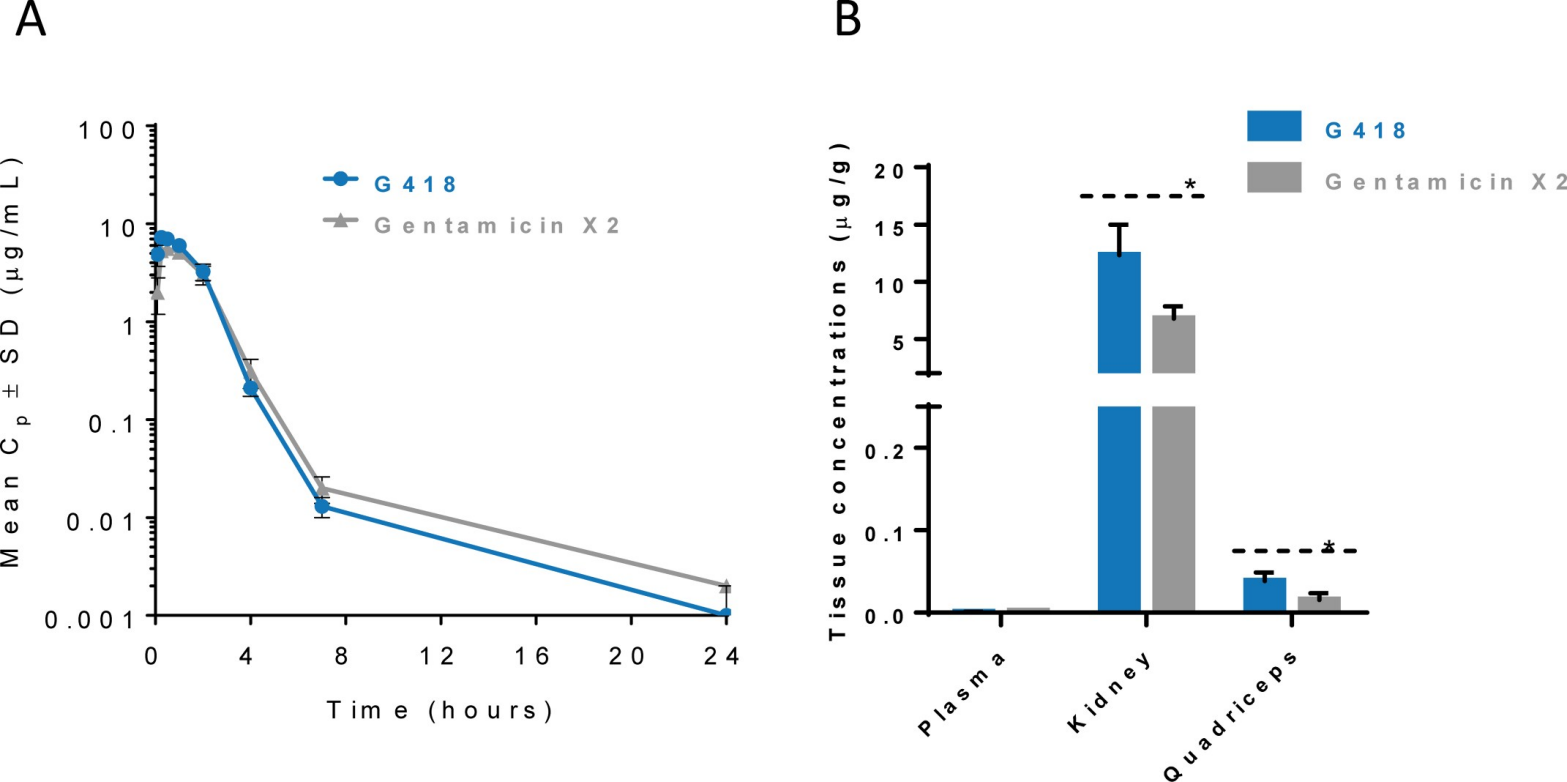
G418 and gentamicin X2 have similar pharmacokinetics in the rat. (A) G418 and gentamicin X2 were administered by subcutaneous injection to rats at a dose of 10 mg/kg. Plasma was collected at the indicated time points and drug levels were determined. Symbols represent the mean of three rats ± standard deviation (SD) per group. (B) G418 and gentamicin X2 accumulate in kidney tissues. The concentration of aminoglycoside in the tissue (μg compound per gram wet weight of tissue) at 24 hours post-dose is the mean ± SD of three rats. Levels of aminoglycosides were at or below the lower limit of quantification. * p < 0.05 (Student’s t-test).

Although G418 has long been known as one of the most active and potent readthrough aminoglycosides due to the combination of its readthrough and mRNA stability functions [24;25;31], evaluation of its in vivo safety has lagged behind. We compared the in vivo safety of gentamicin X2 and G418 because they had the most favorable in vitro readthrough and cytotoxicity profiles (**Fig 2**). We first determined that G418 and gentamicin X2 had similar pharmacokinetic profiles in rats (**Fig 3 and Table 3**). When administered by subcutaneous injection at a dose of 10 mg/kg, G418 and gentamicin X2 showed similar plasma 24-hour area under the curve (AUC_24_), 15 h μg/mL and 13 h μg/mL, respectively (**Fig 3A**). Twenty-four hours after dosing, plasma levels of G418 and gentamicin X2 were at or below the lower limit of quantification (~1 ng/g tissue) and low in the quadriceps (~40 ng/g tissue). Levels were much higher in the kidney (**Fig 3B**), consistent with previous publications demonstrating that aminoglycosides accumulate in the kidney, likely contributing to renal toxicity [39;40]. Kidney levels of G418 were higher than those of gentamicin X2, 12 ± 3 and 7 ± 1 μg/g, respectively.

**Table 3:** Pharmacokinetics parameters.

	G418	Gentamicin X2
T_max_ (hr)	0.33	0.5
C_max_ (μg/mL)	7.4	5.6
AUC_Last_ (h.μg/mL)	15	13
Half-life (hr)	1.5	2.6

**Fig 4:**
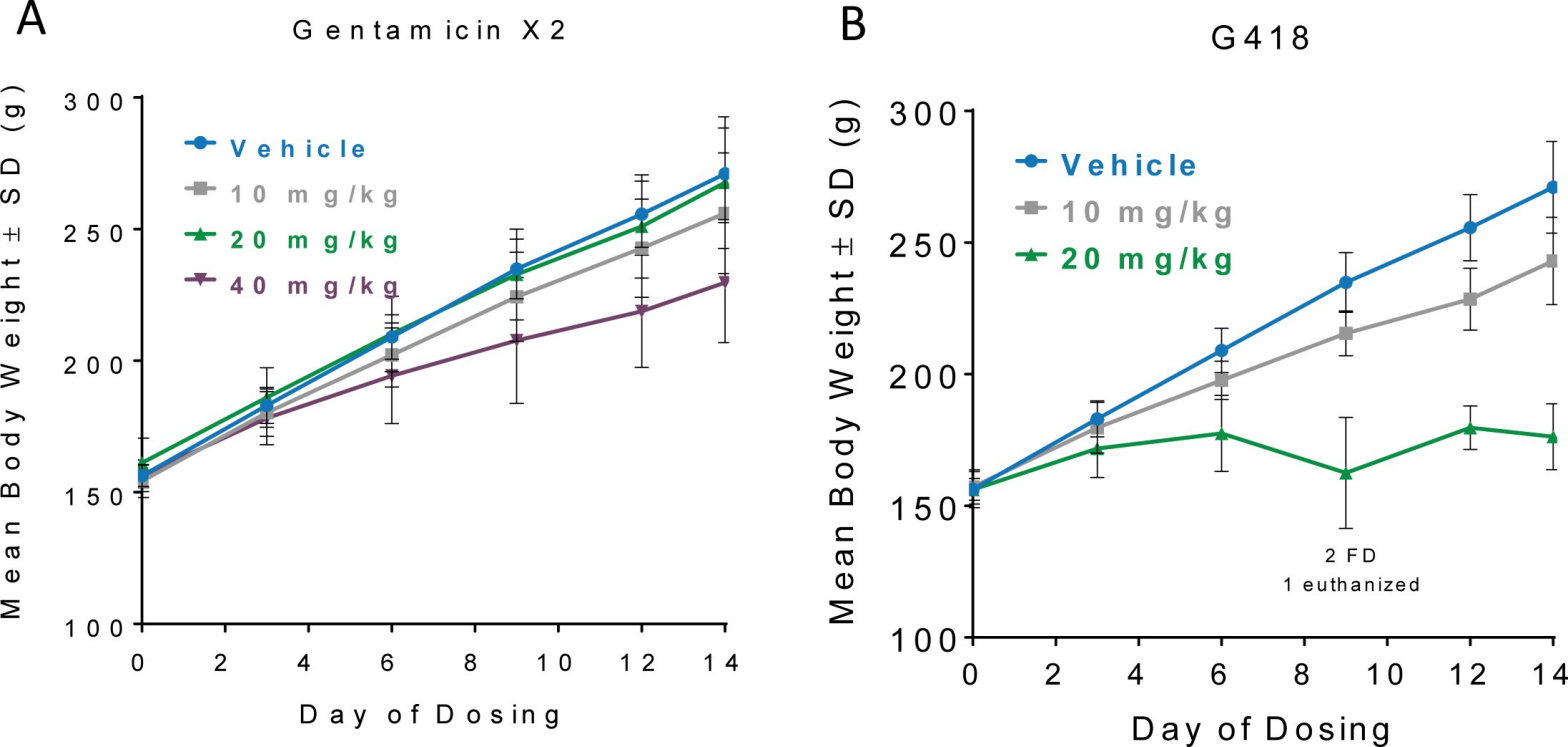
Gentamicin X2 was better tolerated than G418. When administered at a dose of 20 mg/kg G418 (A) caused a greater decrease in body weight than did gentamicin X2 (B) when administered at a dose of 40 mg/kg. Rat body weights can be found in S1 Table.

To evaluate in vivo safety we administered the compounds daily to rats (n = 6 rats per dose group) by subcutaneous injection for 14 days. Toxicokinetics evaluated on Day 1 and Day 14 showed no accumulation of either compound over time (**S4 Fig**). Gentamcin X2 was well-tolerated at all dose levels compared to G418. (**Fig 4**). At the highest dose of G418 (20 mg/kg), three rats were either euthanized or found dead and the rats that tolerated the full 14 days of dosing exhibited significant reductions in body weight -35% less than vehicle treated rats by day 14 (**Fig 4B**). In contrast, gentamicin X2 did not affect body weight at a dose of 20 mg/kg, and only slightly reduced body weight at a dose of 40 mg/kg (**Fig 4A**). No rats were found dead or euthanized in any of the three X2 dose groups.

We also evaluated hematological parameters and found no change in the numbers of red and white blood cells or platelets in any of the gentamicin X2 dosed groups compared to those rats treated with vehicle alone (**S2 Fig**).

### Gentamicin X2 caused less kidney damage than G418

Aminoglycosides are known to cause acute kidney injury in humans (Guthrie 91–96;Wargo and Edwards 573–77). Kidney toxicity is typically measured using creatinine and blood urea nitrogen. On Days 7 and 14, creatinine and blood urea nitrogen (BUN) levels (measures of kidney function) were significantly elevated (p<0.05, one way ANOVA, multiple comparisons vs vehicle) in rats treated with G418 at a dose of 20 mg/kg (**S3 Fig)** indicating that G418 was more nephrotoxic than gentamicin X2.

**Fig 5:**
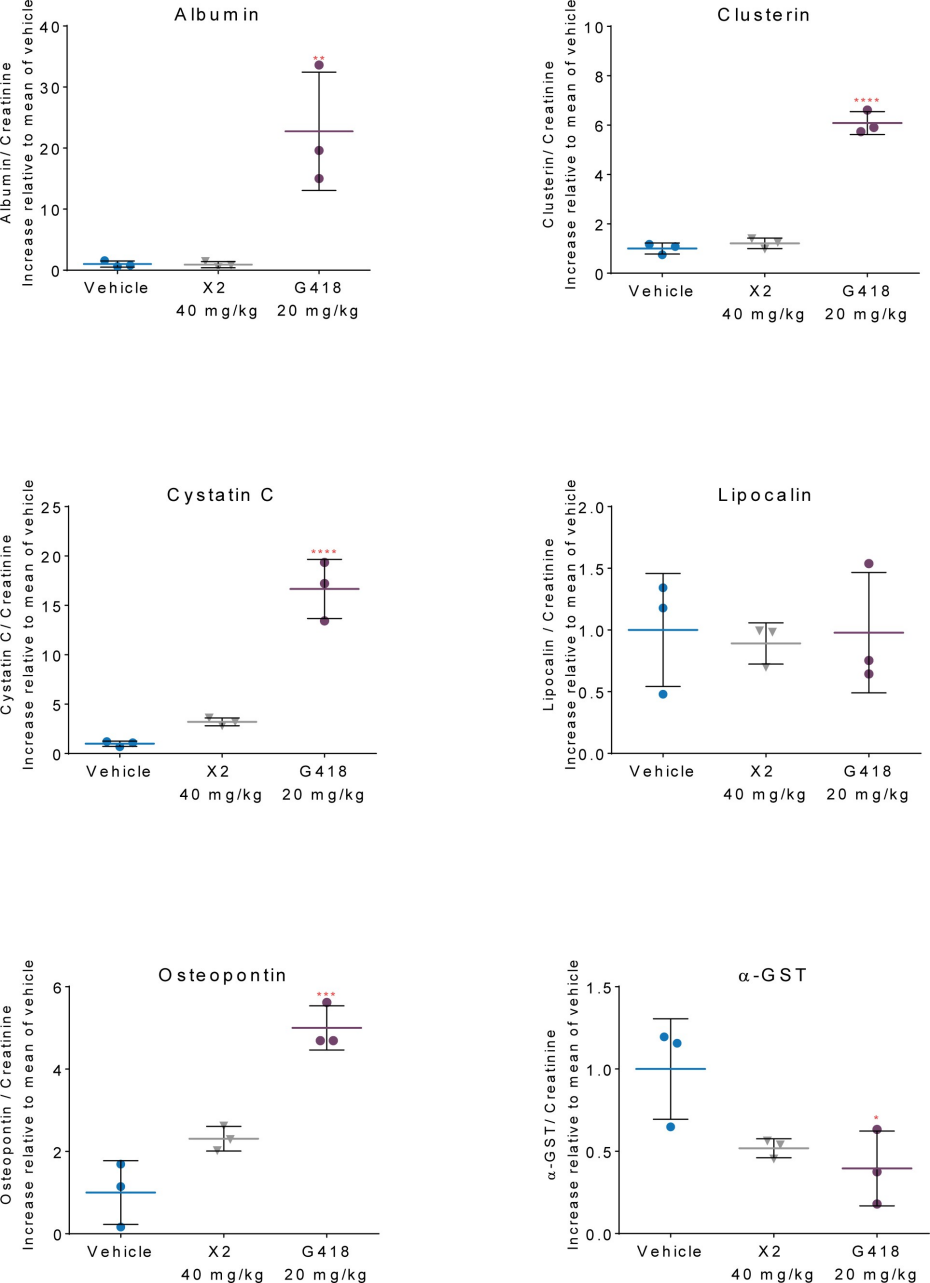
Elevated markers of kidney damage with G418 but not with gentamicin X2. After 9 days of dosing, urine was collected from vehicle treated and the highest dose groups of G418 and gentamicin X2 and analyzed for the indicated markers of kidney damage. Albumin, clusterin cyctatin and osteopontin were increased in the urine of rats dosed with G418 (20mg/kg) but not increased in urine from rats dosed with gentamicin X2 (40 mg/kg). Alpha-GST levelsshowed the greatest decrease in rats dosed with 20 mg/kg G418 compared to 40 mg/kg gentamicin X2. Lipocalin was not affected by either drug.

Urine markers for kidney damage were also analyzed in the highest dose groups (gentamicin X2 at 40 mg/kg and G418 at 20 mg/kg) at day 6 and in the mid-level groups (gentamicin X2 at 20 mg/kg and G418 at 10 mg/kg) at day 9. Albumin, clusterin, cytostatin C and osteopontin levels were all significantly increased in the groups administered G418. The gentamicin X2 group was less affected, indicating that G418 caused more kidney damage at a dose of 20 mg/kg compared to highest gentamicin X2 group (40 mg/kg) (**Fig 5**).

Histopathologic analyses were performed on tissues from the kidney, liver and sternum (for bone marrow) at the end of the 14 day dosing period. Histopathology analyses of kidneys from all rats administered G418 at a dose of 20 mg/kg showed tubular necrosis and pelvic dilation. Histopathology analyses of kidneys from rats administered gentamicin X2 at a dose of 40 mg/kg revealed less damage (four of six kidneys had tubular necrosis and pelvic damage) compared to rats administered G418 (**Table 4**). No histopathology findings were observed in the liver or bone marrow from rats dosed with either gentamicin X2 or G418. Thus, consistent with the urine analysis, rats administered G418 at a dose of 20 mg/kg had more damage to their kidneys than rats administered gentamicin X2 at a dose of 40 mg/kg.

**Table 4:** Rat kidney histopathology.

	Vehicle	X2 (10 mg/kg)	X2 (20 mg/kg)	X2 (40 mg/kg)	G418 (10 mg/kg)	G418 (20 mg/kg)
Animals completed study	6	6	6	6	6	3
Animals examined	6	6	6	6	6	3
*Kidneys*						
Kidneys examined	6	6	6	6	6	3
Mineralization	0	0	0	0	1	0
Tubular necrosis	0	2	2	4	4	3
Pelvic dilation	0	0	1	4	2	3

## Discussion

Gentamicin is a complex of multiple major and minor components. The readthrough activity, potency, toxicity, and the relative abundance of the individual gentamicin components contribute to overall potency and activity. Greater than 92% of the gentamicin complex is composed of gentamicins C1, C1a, C2, and C2a [26–28]. We found, as expected, that potency of the complex (EC_2X_ = 418 μM ± 310), was similar to that of the major complex components (EC_2X_ range of 142–311 μM) (**Table** 1). It has been shown recently that gentamicin B1, an unquantified minor component of the complex, has considerably greater readthrough activity and potency than the complex or any of its major components [41]. Although we were unable to obtain gentamicin B1 for testing, we present data showing that another minor component, X2, is also a potent and active readthrough aminoglycoside. The amount of B1 and X2 likely vary between gentamicin preparations and it was therefore proposed that the varying amounts of highly potent complex components could cause variable readthrough activity of different gentamicin preparations [41]. However, because the readthrough activity and potency of the major individual components are similar in magnitude to that of the complex (**Table 1**), it is also possible that the major components are responsible for the bulk of the readthrough activity of the complex.

**Fig 6:**
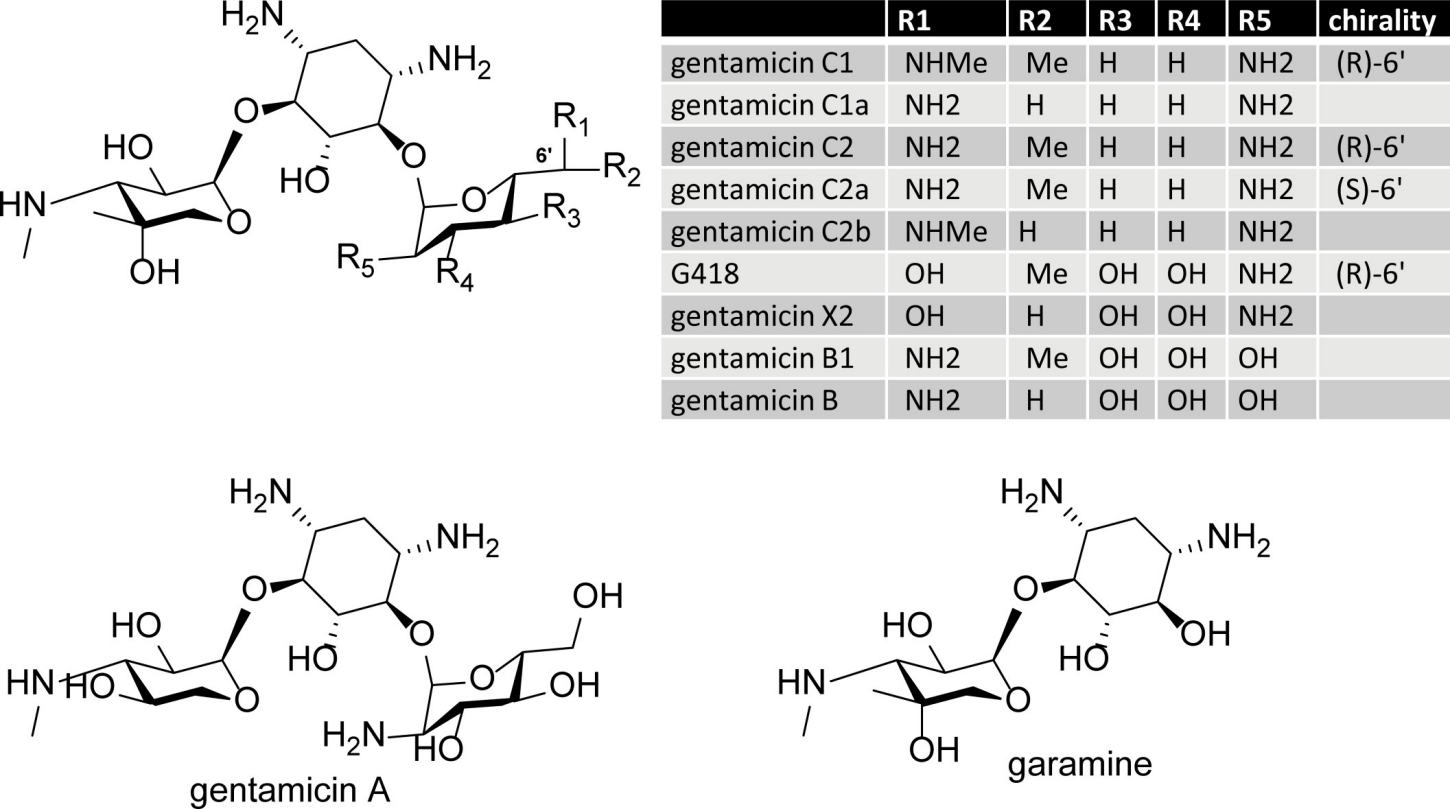
Structure of G418 and gentamicin complex components. The structure on the top left and the table show the structural differences between gentamicin complex components and G418. The structure of the other two gentamicin complex components are shown in the lower two drawings.

As reported previously [24;25], and confirmed here, G418 is the most potent aminoglycoside for readthrough activity. It differs structurally from the majority of gentamicin components by having a hydroxyl group at the C(6’) position of ring A (**Fig 6**). The only structural difference between G418 and X2 is the replacement of the C(7’) carbon atom with a hydrogen atom in gentamicin X2. G418 is an isomer of gentamicin B1 in which the OH and NH_2_ groups at C(2’) and C(6’) are switched. It is thought that the C(6’)-OH group is crucial for readthrough activity in eukaryotic organisms [26;42]. Recent structural work with G418 bound to ribosomal RNA [36] [14;36] indicates that the C(6’)-OH group (H-bond acceptor) of G418 and the exocyclic NH_2_ group (H-bond-donor) of ribosomal guanosine at position 1645 form a hydrogen bond. This hydrogen bond is believed to be crucial for the specific binding of G418 to the eukaryotic ribosome. If the C(6’)-OH is replaced by a C(6’)-NH_2_ group, as it is in the major components of the gentamicin complex, the NH_2_ group will be highly protonated [43] at physiological pH and thus, unable to act as an efficient H-bond acceptor. This likely explains the lower eukaryotic readthrough potency of the gentamicin complex, which consists predominantly of gentamicins C1, C1a, C2, and C2a, each containing a C(6’)-amino group.

Others have prepared and evaluated a series of semisynthetic aminoglycosides and determined that in addition to the nature of the polar group (amine vs hydroxyl) at C(6’) the presence and configuration of the C(6’)-Me group are important for readthrough activity [44]. The most potent compounds in this series contain a C(6’)-methyl group in the (*R*) configuration at C(6’), in addition to the C(6’)-OH group. The same moiety, a secondary alcohol in the *R*-configuration at C(6’), is present in G418.

Aminoglycosides have been investigated as potential readthrough therapeutics. Gentamicin has been studied both in preclinical and clinical settings [18–22]. Designer aminoglycosides (for example, NB124) have also been synthesized and shown to have readthrough activity with reduced cytotoxicity [45–47]. Recently G418 was shown to be more potent than NB124 in readthrough of five different p53 nonsense codons [23]. This is in agreement with our data (**Table 1 and Fig 2**) showing that G418 is more potent than NB124. We evaluated neuromast toxicity (CC_50_) as a surrogate for ototoxicity and found that the CC_50_ values for gentamicin, NB124, NB84, and gentamicin X2 were 2.8 ± 1.2 μM, 15 ± 4 μM, 8 ± 0.1 μM and 14 ± 7 μM, respectively (**Table 2**). This compares favorably with CC_50_ values obtained previously in mouse cochlear explants where gentamicin, NB124, and NB84 had a CC_50_ values of 3.5 μM, 15 μM and 20 μM, respectively [48]. Although the absolute values differ slightly, the rank order is the same indicating that both assays can be used to rank order the cytotoxicity of compounds.

When considering a compound for readthrough therapy, a sufficient ratio of safety to readthrough potency is critical. Here we show that gentamicin X2 has a greater readthrough-safety window than the other compounds evaluated: NB124, NB84 or G418. Our data demonstrate that gentamicin X2 warrants further investigation to determine its potential clinical utility in treating genetic diseases and cancers caused by nonsense mutations.

## Materials and methods

### Animal usage

All in-life animal procedures were performed in a laboratory certified by the American Association for the Accreditation of Laboratory Animal Care (AAALAC) with approval from the Rutgers Institutional Care and Animal Use Committee.

### Aminoglycoside compounds

All compoundssed in this study were obtained from TOKU-E (Bellingham, Washington), except for G418, which was obtained from GoldBio Technology (www.goldbio.com).

### Protein detection

Western blot hybridization and Meso Scale Discovery (MSD) immunoassay detection of p53 were done as previously described [12]. Detection of tGFP-CFTR_542X_-HA-HIS protein expressed from the Turbo GFP-CFTR-G542X construct [31] stably incorporated into 293H cells was done using standard MSD immunoassay methods (Rockville, Maryland, https://www.mesoscale.com). Cells (45K per well) were grown in 96-well plates and treated with compounds for 24 hours. Multi-array 96 well plates (MSD, L15XA) were coated with 2 μg/well HA-tag antibody (Invitrogen, 26183) in 30 μl of PBS (phosphate buffer saline, pH 7.6 and 137 mM NaCl) followed by washing three times with PBS-T (PBS with 0.1% tween-20) and blocking for 1 hour with 150 μl of TBS-C (50 mM Tris pH 7.5, 238 mM NaCl, 2.7 mM KCl and 1% Casein). Cells were lysed with 60 μl per well of lysis buffer (20 mM Tris-HCL, pH 7.5, 1 mM EGTA, 1 mM EDTA,150 mM Nacl, 0.5% Triton X100) for one hour at room temperature and 30 μl of lysate per well were transferred to the coated plate and incubated overnight at 4 ^o^C. Plates were then washed three times with PBS-T and 30 μl per well of 0.25 μg/ml anti-His-tag antibody (Genescript, A00174) in TBS-C was added for 1 hour at room temperature. Plates were then washed three times with PBS-T and 30 μl of 0.25 μ/ml goat anti-rabbit IgG-Sulfo-tag antibody (MSD) in TBS-C was added and incubated for 1 hour at room temperature. Plates were then washed three times with PBS-T and 150 μl read buffer (MSD) was added. Plates were read immediately in a SECTOR S 600 (MSD).

### In vitro fLuc-190-NS readthrough assay

This cell-free translation assay used synthetic LUC mRNAs harboring an EMCV IRES and LUC codon 190-UGA premature stop codon prepared using the MegaScript in vitro transcription kit (Ambion). Translation reactions were done with the Rabbit Reticulocyte Lysate System (Promega) with 100 ng RNA and increasing concentrations of aminoglycosides. The amount of luminescence produced was determined after approximately 4 hours using a Viewlux CCD imager (Perkin-Elmer). Fold suppression over background was calculated as (aminoglycoside light units/PBS light units) [7].

### Cytotoxicity assays

Frozen human Peripheral Blood Mononuclear Cells (PBMCs) were obtained from ALLCELLS (Alameda, CA, Cat# PB003F) from a single donor. Cells were seeded at 50,000 cells per well in a 96-well plate, in RPMI-1640 medium with 10% fetal bovine serum, PHA (REMEL Cat# R308528701 at 20 μg/mL) and IL-2 (BD Pharmingen, Cat#554603 at 40 ng/mL). Plates were incubated at 37°C with 5% CO_2_ for 4 hours prior to compound treatment. Compounds were serially diluted and added to the plates. Plates were incubated at 37°C, 5% CO_2_ incubator, for 72 hours prior to determinations of ATP levels. Cell viability was determined using a chemiluminescent assay to monitor adenosine triphosphate (ATP) levels in live cells (CellTitre-Glo; CTG; Promega, Cat#G7571). Methods were performed per the manufacturer’s instructions.

### Neuromast toxicity assays

All neuromast cytotoxicity assays were done at Biobide–BBD Biophenix SL (Gipuzkoa, Spain). Zebrafish embryos 5 dpf (days post fertilization) were exposed to the drug in 24-well plates, 5 embryos per well, two wells per condition to have a sample size of ten embryos. Compounds were tested at 5 doses. After 24 hours of exposure embryos were incubated in DASPEI (2-(4-(dimethylamino)styryl) -N-Ethylpyridinium Iodide) for neuromast staining. Images of each embryo were taken and neuromasts counted. A compound was classified as ototoxic when the number of neuromasts in the treated group was less than that in the untreated control group (One Way ANOVA, multiple comparisons vs. vehicle). The CC_50_ was defined as the concentration required to reduce the number of neuromasts by 50%.

### Pharmacokinetics of aminoglycosides in the rat

Test compounds were dissolved in PBS at 1 mg/mL and administered subcutaneously in a volume of 10 mL/kg to deliver a dose of 10 mg/kg. For each compound, 3 rats were dosed and blood was obtained by retro-orbital bleeding at 0.083, 0.25, 0.5, 1, 2, 4, 7, 24 and hours post-dose. At 24 hours, the rat was euthanized and in addition to blood, quadriceps and kidney tissues were collected for subsequent analysis.

### Fourteen day safety study in rats

Male Sprague Dawley rats were purchased from Charles River Laboratories, where 6 weeks old at time of study initiation. Rats were group-housed (3 rats per box) in solid bottom cages. Food and water were provided ad libitum. Rats were dosed daily by subcutaneous injection. Groups included 9 rats dosed with vehicle and 6 rats per dose group for gentamicin X2 or G418. Blood was collected for serum chemistry by retro-orbital bleeds on Day 7 and by terminal cardiac puncture on Day 14. For urine collection, rats were housed in metabolic cages for 6 hours. Toxicokinetics was evaluated on Day 1 and Day 14 (**S4 Fig**). Blood was obtained by retro-orbital bleeding at 0, 0.25, 0.75, 1.5, 4, 7, 24 hours post-dose. Rats were euthanized on Day 14, 24 hours after the last dose. Body weights were measured on Days 3, 6, 9, 12 and 14. Endpoints to evaluate kidney function included serum chemistry (BUN and creatinine; Day 7 and Day 14), urine biomarkers (albumin, clusterin, cystatin C, osteopontin, KIM1, and alpha GST; Day 6 and Day 9), hematology (Day 14) and histopathology. For histopathology, kidney, liver, and bone marrow were evaluated. Samples collected for histopathology were sectioned at 5 μm, stained with hematoxylin and eosin (H&E), and examined by a board certified veterinary pathologist. BUN, creatine and hematology parameters were measured using a HESKA veterinary system (Loveland, CO). Urine samples were assayed using MSD electrochemiluminescent immunoassay. Samples were analyzed in triplicate using a Sector Imager 6000 instrument (MSD, Rockville, Maryland). Kits used were the Rat Kidney Injury Panel 1 (albumin, Kim-1, NGAL/lipocalin-2, osteopontin), the Argutus Acute Kidney Injury Panel (α-GST, GSTYb1, RPA-1) and the Clusterin Test Kit (MSD), according to the manufacturer’s instructions. Urinary biomarker concentrations were normalized to urinary creatinine to account for differences in urine production and flow rates [49].

### Compound quantification

Blood was collected into tubes containing dipotassium ethylenediaminetetraacetic acid (K2 EDTA) as the anticoagulant and rotated at room temperature until the blood was centrifuged and the plasma recovered for subsequent analysis by liquid chromatography with tandem mass spectroscopy (LC-MS/MS). Noncompartmental toxicokinetic parameters were determined using Phoenix WinNonLin version 6.1 (Pharsight Corporation, Carey NC).

The concentrations of test compound in plasma, kidney and muscle were quantified by liquid chromatography-tandem mass spectrometry (LC-MS/MS). Briefly, the plasma and tissue homogenate samples were treated with acetonitrile-methanol mixture containing an internal standard that is a close analog of the test compounds. The treated plasma and brain homogenate samples were centrifuged and the supernatant was collected and analyzed using electro-spray LC-MS/MS.

### Statistical methods

Readthrough activity and toxicity measurements were analyzed by means of a general linear model. The Tukey-Kramer studentized range test was used to compare all main effect means while maintaining intended alpha levels [32]. Safety windows were calculated by dividing the average toxicity measurements by the average readthrough measurements. Fieller’s theorem was applied to calculate 95% confidence limits on these ratios [33]. Statistical analysis for this paper was generated using SAS/STAT software, Version 9.4 of the SAS System for Windows (SAS Institute Inc.)

## Supporting information

S1 Checklist NC3Rs ARRIVE Guidelines Checklist.pdf. (PDF)

S1 File All readthrough and cytotoxicity data used in this study. (XLSX)

S1 Fig Neuromast cytotoxicity. Zebrafish larvae at 5 days post fertilization were incubated with increasing concentrations of aminoglycosides for 24 hours. The box and whiskers bars show the median ± minimum value to maximum value for 10 replicates.
(EPS)

S2 Fig Administration of G418 and gentamicin X2 does not overtly deplete red blood cells, hemoglobin, white blood cells, or platelets. Rats were dosed by subcutaneous injection for 14 days with G418 or gentamicin X2 and euthanized approximately 24 hours after the last dose. Complete blood counts were evaluated with no obvious differences across groups. Each symbol represents values from an individual rat. The mean is shown as the line for each group.
(EPS)

S3 Fig Administration of G418 (20 mg/kg) increases BUN and creatinine levels. Symbols represent values for individual rats (6 rats per group). For Day 7, all groups included 6 rats per group. For Day 14, in the group dosed with G418 (20 mg/kg), there were 3 rats surviving. Also included in this group (shown in lighter green, highest values in the group) are data from one rat that was euthanized on Day 12 so that there are 4 symbols shown. For Day 14, all other groups included 6 rats per group.
(EPS)

S4 Fig Toxicokinetics of G418 and gentamicin X2. Compounds levels were determined from retro-orbital bleeds at the indicated time points. Error bars represent standard deviation of the mean from two rats. No accumulation was observed between day 0 and day 13.
(EPS)

S1 Table Rat body weight for 14 day safety study. (EPS)
